# Ups and downs in early electron cryo-microscopy

**DOI:** 10.1371/journal.pbio.2005550

**Published:** 2018-04-19

**Authors:** Jacques Dubochet, Erwin Knapek

**Affiliations:** 1 Department of Ecology and Evolution (DEE), University of Lausanne, Lausanne, Switzerland; 2 Bundesverband Geothermie, Berlin, Germany

## Abstract

This is a tale of two scientists who, in their younger days, had their scientific judgement clouded by the promise of a big discovery. Two years later, they found that their conclusions had been considerably exaggerated. They were lucky, though, as their later work would prove to be significant. Now, more than 30 years after those events, they met again and put in writing their understanding of what went wrong.

It was the 1970s; physicists, engineers, and biologists were together exploring how the resolution achievable on biological specimens with an electron microscope might be improved by operating at a low temperature. Could water evaporation be avoided? Could the damaging effect of the electron beam be reduced? For the latter question, it was found that the temperature of liquid nitrogen (88 K) provides moderate cryoprotection, but very low temperatures—in the range of a few Ks—had barely been explored (see Box 1).

**Box 1. Why electron cryo-microscopy?**

The major limitations of electron microscopy are due to (i) the damage that high-energy electrons inflict on the specimen and, for biologists, (ii) the fact that liquid water, the most abundant constituent of biological material, cannot be retained in the high vacuum of the microscope. Since the early 1960s, electron cryo-microscopy had been considered a potential route to overcoming both of these limitations [1]. For instance, one might imagine that beam damage would be reduced at low (88 K, boiling liquid nitrogen) or very low (4 K, boiling liquid helium) temperatures; indeed, later results have confirmed this impression, albeit to a limited extent. The present article relates to a report in the early 1980s that exaggerated the advantage of very low temperatures [2].

Around the same time, it was found that biological specimens can be vitrified for electron cryo-microscopy observations. Under such conditions, water is immobilised in a vitreous state in which biological structures appear perfectly preserved [3]. This finding opened the avenue that led—three decades later and through the contribution of many scientists (those of Richard Henderson and Joachim Frank were of decisive importance)—to major progress in molecular structural biology and to the 2017 Nobel Prize in Chemistry.

In 1979, the group of Isolde Dietrich from Siemens AG in Munich, where Erwin Knapek was a research scientist, announced that beam damage is ‘dramatically reduced’ when the specimen is observed close to liquid helium temperature [4]. They obtained this result in a newly developed electron cryo-microscope equipped with a superconducting lens operating at 4.2 K with unprecedented electrical, mechanical, and thermal stability. The result, however, was still to be rigorously quantified.

Before this result was published, rumour had reached Jacques Dubochet. A postdoc with Édouard Kellenberger in Basel at the time, Jacques had just been hired as group leader by Sir John Kendrew, director of the newly formed European Molecular Biology Laboratory (EMBL) in Heidelberg. His project was aimed at exploring water and hydrated biological specimens by electron cryo-microscopy. During his PhD, in the steps of Bob Glaeser [5], Jacques had become experienced in the quantitative measurement of beam damage. Very excited, he called Erwin in Munich: ‘You have a phenomenal instrument, and I know how to measure quantitatively its extraordinary cryoprotection effect; we should join forces’. So that is what they did during Jacques’ last few months in Basel and in the first months of his time in Heidelberg. It was a great time for both of them.

Jacques would travel on the early train from Basel or Heidelberg, bringing the freshly prepared specimen to Erwin and his complicated microscope in Munich. The instrument had little in common with the easy-to-use electron microscopes we know today. Part of the challenge was that while the superconducting current was perfectly stable, it could not be changed easily; focusing relied on varying the high voltage. Switching from direct imaging to electron diffraction was also quite an adventure, without even considering the initial cooling of the lens and the process of aligning it. Erwin was the master of the operation, Jacques the master of specimen preparation. Erwin struggled in the dark for hours while Jacques, sitting behind, looked over his shoulder.

Most of the work consisted of recording the evolution of electron diffractograms of organic crystals under increasing electron dose. The results soon emerged, and for Jacques and Erwin (Fig 1), they were clear. They were documented by a series of diffractograms from which the cryoprotection factor compared to room temperature could be determined. It was in the range of 30 to 300—a groundbreaking result for electron microscopy. These results were reported in the *Journal of Molecular Biology*, under the title ‘Beam Damage to Organic Material is Considerably Reduced in Cryo-electron Microscopy’ [2].

**Fig 1:**
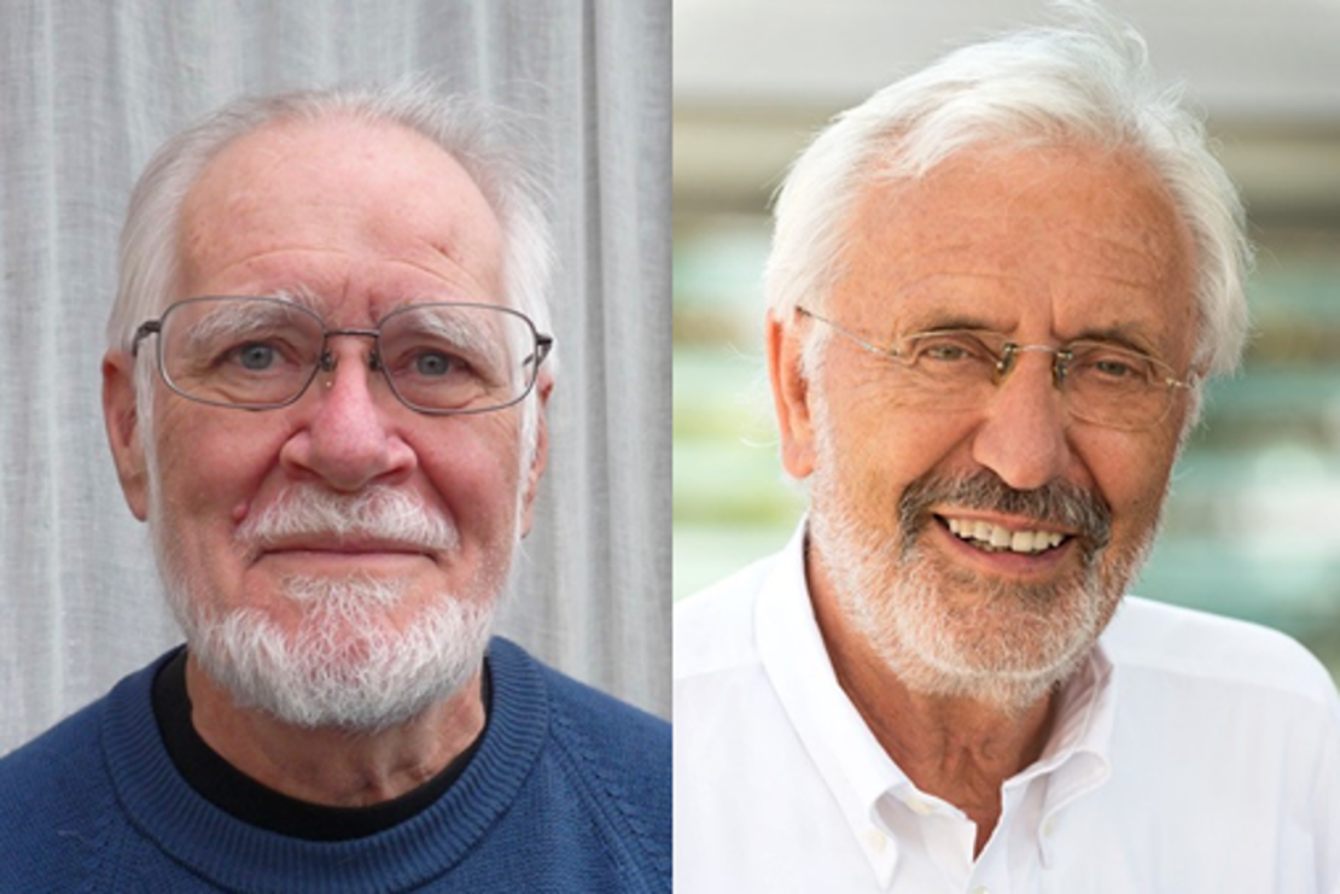
Recent photos of the authors. Left: Jacques Dubochet. Right: Erwin Knapek. From personal collections.

With such striking results, it was easy for Jacques to convince the director of EMBL to order another lens, and to instruct Arthur Jones, head of the electron microscopy development group, to customise an existing microscope. This took longer than planned, but two years later, Jacques and his team were ready. Jean Lepault, a French scientist experienced in cryo-specimen preparation, had joined the group, and together they began reproducing the results on a microscope that was much easier to use than the original one—so much easier, in fact, that one week later, Jacques was back in the director’s office reporting that ‘We observe no cryoprotective effect, or at best, a very minor one’. Kendrew replied, ‘Ah, make sure of it, and make it known!’

So appeared a note in *Nature* [6] and an 18-line ‘Amendment’ to the original article stating that they were unable to reproduce the previous results [7]. A collaborative reevaluation by nearly all the laboratories involved in electron cryo-microscopy of biological specimens at very low temperature [8] was underway. It concluded that, compared to what happens at the temperature of liquid nitrogen, ‘It is not possible to say with any certainty that a further significant change in cryoprotection is to be observed [close to] 4 K’.

In other words, the claims of ‘dramatic’ or ‘considerable’ reduction of beam damage at very low temperature [2] had been much exaggerated. What were the reasons for this mistake?

When discussing our erroneous result in public, we have sometimes suggested a plausible explanation. Electrical and thermal conductivity decrease with temperature, thereby favouring accumulation of charge and heat in the specimen and increasing its tendency to drift. Under conditions in which the electron diffractogram is observed (as opposed to the image), it is possible that movement of the specimen could constantly expose new regions to the electron beam without being noticed.

We never claimed these explanations were entirely adequate, and we recognise that we should have been more self-critical. We now realise that the error had another less obvious root, a root that can be found in Popper’s old idea of falsification [9], which requires that a scientist with a good idea must make every effort to prove it wrong. It is only the failure of this effort to falsify which supports the hypothesis. This approach is admirable but is not the way that people naturally think; anyone with a good idea surely wants to prove it right, not wrong. Accordingly, many scientists spend years trying to prove a controversial idea; sometimes they are right, sometimes they fail. That’s how science goes.

It is fair to say, though, that the experimental procedure involved was delicate and the physical system was complex. The conclusions of the International Study Group [8] explained the difficulty of controlling all the relevant experimental parameters. In particular, attention had to be paid to the chemical reactions taking place in the specimen, and to its real temperature. The latter could depend on factors such as the temperature of the specimen holder, the thermal and electrical conductivity of the specimen, the geometry of the support, the dose rate, and so on. Reanalysing the data of the time [10], it was suggested that the thermal contact between the specimen and the supporting film was of upmost importance. This would help explain how results from Munich were corroborated independently in a similar system operating in Berlin [11]. Whatever the source of the error, understanding beam damage at very low temperature still needed more work.

We had every reason to love the words ‘dramatic’ or ‘considerable’ because these adjectives would have perfectly characterised the breadth of our results—if they had been correct. They would have marked a leap forward for electron microscopy, and they would have secured the continuation of our careers in uncertain times. However, our drive to succeed, our ambition, and our pride clouded our scientific judgement. The distinction between creative thinking and biased interpretation is a narrow one. We erred, but later, we were lucky to be able to identify the error and correct our mistake. In subsequent years, we also had the opportunity to be successful in different ways. Not everyone will be so lucky. Young scientists, learn from our error! It is not easy to be a good scientist, but it can be learned.

It took us 35 years to write this candid report and a Nobel Prize to make it acceptable for publication. Science would benefit from recognising that the path of creativity is narrow for everybody. Constructive reporting—and active discussion—about slip or failure could help prevent others from straying.

Jacques Dubochet is an honorary professor of biophysics at the University of Lausanne, Switzerland.

Erwin Knapek is president of the German Geothermal Society, Berlin and Augsburg.
